# The roots of healthy aging: investigating the link between early-life and childhood experiences and later-life health

**DOI:** 10.1186/s12877-023-04297-z

**Published:** 2023-10-10

**Authors:** Nan Lu, Peng Nie, Joyce Siette

**Affiliations:** 1 Renmin University of China, Beijing, China; 2 Xi’an Jiaotong University, Xi’an, China; 3 The MARCS Institute for Brain, Behaviour and Development, Western Sydney University, Sydney, Australia

## Abstract

Whilst early-life conditions have been understood to impact upon the health of older adults, further exploration of the field is required. There is a lack of consensus on conceptualising these conditions, and interpretation of experiences are socially and culturally dependent.

To advance this important topic we invite authors to submit their research to the Collection on ‘The impact of early-life/childhood circumstances or conditions on the health of older adults’.

In recent years, there has been a growing trend among social scientists and public health researchers to employ life course data and analytical techniques as means of better comprehending the biological, social and environmental factors that determine health outcomes during the later stages of life. By tracing the association between social circumstances and health over the course of an individual’s life, from childhood through to older age, this approach seeks to develop a more nuanced understanding of this complex relationship.

The importance of early life experiences on people’s health throughout the life-course is not novel. Decades of research have identified the impact of early life experiences on later health [1]. Indeed, recent studies have found a number of relevant childhood variables, including but not limited to socioeconomic status, adverse experiences (e.g., abuse and neglect), disease, and health resources during childhood [2], with cascading effects on health during adulthood and late adulthood. Proponents of the latency model suggest that poor childhood conditions could have a long-term and irreversible influence on individuals’ health trajectories patterns [3]. For example, malnutrition in childhood could weaken immune systems and contribute to lower growth rates of musculoskeletal systems, which could further influence joint inflammation in later life [4].

Adverse experience and poor health care resources in childhood could also impose a long-term adverse impact on brain development, which could contribute to cognitive impairment at older age [5]. Furthermore, the pathway model suggests that childhood conditions could indirectly affect health in later life through adulthood conditions [1]. Life-course perspective and cumulative inequality theory have further enriched our understandings of protective and risk factors in early life and how they affect the health of older adults [3].

Given that the first 1000 days of life between conception and a child’s second birthday have short and long-term effects on human health and function, and are identified as the most crucial window of opportunity for interventions [6], a growing number of studies have investigated the linkage between in utero circumstances and health in later ages.

From a life-course perspective, the fetal origins hypothesis posits that fetal exposure to an adverse environment, in particular to in utero malnutrition, is associated with increased risks of cardiovascular and metabolic diseases of adults and older ages [7]. A large body of literature has validated this hypothesis in older populations. For instance, a strand of existing studies has linked fetal malnutrition or famine with later-life health problems, including decreased glucose tolerance, schizophrenia, heart disease, obesity, type 2 diabetes, increased mental illness and mortality. Additionally, prior literature also associated other in-utero risk factors such as exposure to conflict and violence [8], and influenza pandemic [9], with ill health in older age.

Nonetheless, life-course studies on the nexus between prenatal adversities and later health suffer from threats from mortality selection [10]. As such, the early-life impacts of adversities on later-life health may be weak or even disappear when the influence of selection outweighs the detrimental effects of fetal exposure to adversities or fetal exposure indeed has no long-run health impacts [10]. We acknowledge that estimates of the effects of in utero exposures on later-life health may be sensitive for different analytic approaches and measures of health outcomes.

Although there are a number of studies concerning the “long-arm” of childhood conditions, there remains major research gaps. First, the interpretations of childhood experiences are culturally and socially dependent. Therefore, empirical evidences across countries and culture, especially those from developing countries and regions, are needed to test these hypotheses.

Additionally, there is a lack of consensus on the conceptualization and measurement of childhood conditions. While many studies assess one or several aspects of childhood conditions, future studies are recommended to use a comprehensive set of measures of childhood conditions to test their combined effects on an individual’s health in later life simultaneously. Indeed, the exploration of multiple experiences and exposures will enable a better assessment of the breadth of childhood adversity and opportunities and its link with both adults’ and older adults’ health.

Longitudinal research allows us to not only test the baseline level (i.e. intercept) and change rate (i.e. slope) of health outcomes and how they were affected by childhood conditions, but also examine the mechanisms linking childhood conditions, adulthood conditions, and health outcomes in later life.

Enhancing our comprehension of the cumulative impact of childhood experiences across various key timepoints can promote multidisciplinary prevention strategies that emphasize early intervention. By providing collaborative services that address diverse adversities affecting individuals and families throughout their lives, these efforts can deliver integrated programs that offer support and decrease the likelihood of future generations being impacted by negative experiences.

Optimizing the long-term health of individuals requires an in-depth understanding of the roots of healthy aging, from early experiences to mid-life health, and its associated impact on later-life health. Physical, social, mental and biological environments are likely to play a synergistic, critical, yet complex role in promoting and maintaining healthy aging. In this Collection, we aim to present original research and evidence synthesis to advance our understanding of the relationship between early experiences, later-life health, and the physical, social, and organizational aspects of being. We particularly welcome contributions that explore this relationship and offer insights into optimizing aging and wellbeing. We hope that this collection will empower healthcare professionals, researchers and policy makers to find innovative ways to enhance care and promote healthy aging on a population-level.

## Data Availability

Not applicable.
